# Wear-Time Compliance with a Dual-Accelerometer System for Capturing 24-h Behavioural Profiles in Children and Adults

**DOI:** 10.3390/ijerph15071296

**Published:** 2018-06-21

**Authors:** Scott Duncan, Tom Stewart, Lisa Mackay, Jono Neville, Anantha Narayanan, Caroline Walker, Sarah Berry, Susan Morton

**Affiliations:** 1 School of Sport and Recreation, Auckland University of Technology, Auckland 1142, New Zealand; tom.stewart@aut.ac.nz (T.S.); lisa.mackay@aut.ac.nz (L.M.); em10257@aut.ac.nz (A.N.); 2 School of Engineering, Computer and Mathematical Sciences, Auckland University of Technology, Auckland 1142, New Zealand; jono.neville@aut.ac.nz; 3 Centre for Longitudinal Research, University of Auckland, Auckland 1142, New Zealand; caroline.walker@auckland.ac.nz (C.W.); sarah.berry@auckland.ac.nz (S.B.); s.morton@auckland.ac.nz (S.M.)

**Keywords:** physical activity, sedentary behaviour, measurement, accelerometry, motion sensors, time-use epidemiology

## Abstract

To advance the field of time-use epidemiology, a tool capable of monitoring 24 h movement behaviours including sleep, physical activity, and sedentary behaviour is needed. This study explores compliance with a novel dual-accelerometer system for capturing 24 h movement patterns in two free-living samples of children and adults. A total of 103 children aged 8 years and 83 adults aged 20-60 years were recruited. Using a combination of medical dressing and purpose-built foam pouches, participants were fitted with two Axivity AX3 accelerometers—one to the thigh and the other to the lower back—for seven 24 h periods. AX3 accelerometers contain an inbuilt skin temperature sensor that facilitates wear time estimation. The median (IQR) wear time in children was 160 (67) h and 165 (79) h (out of a maximum of 168 h) for back and thigh placement, respectively. Wear time was significantly higher and less variable in adults, with a median (IQR) for back and thigh placement of 168 (1) and 168 (0) h. A greater proportion of adults (71.6%) achieved the maximum number of complete days when compared to children (41.7%). We conclude that a dual-accelerometer protocol using skin attachment methods holds considerable promise for monitoring 24-h movement behaviours in both children and adults.

## 1. Introduction 

It is well established that regular physical activity, limited sedentary time, and adequate sleep are essential for optimal health and wellbeing [1,2,3]. Studies have largely investigated these distinct but strongly interrelated behaviours as independent entities; however, accumulating evidence suggests that different combinations of time spent in these behaviours may impact health in ways that cannot be explained by studying each behaviour in isolation [4,5]. This has prompted a global paradigm shift where an integrated movement approach focusing on complete (24 h) days is now a research priority, a movement called time-use epidemiology [6]. Indeed, public health agencies in Canada (followed by New Zealand and Australia) recently released universal movement guidelines that provide physical activity, sedentary behaviour, and sleep recommendations across a hypothetical 24 h day [7,8].

Despite the advantages of considering all behaviours along the movement continuum, objectively, capturing free-living data round-the-clock is notoriously difficult [9]. Researchers have relied on hip-mounted motion sensors (accelerometers) for the last decade but this method has several significant limitations. When placed on the hip, these devices are unable to accurately differentiate between sitting, lying (sleep), and ‘non-wear time’ [10,11]; a critical limitation given that unfavourable sleep and sedentary patterns are thought to activate discrete pathophysiological pathways and are thus distinct components of a 24 h composition [12,13,14]. This also raises the burden of analysis because each day of wear for each participant has to be analysed separately to ‘remove’ the sleep period before physical activity and sedentary behaviour can be properly examined [14]. Participant compliance to hip-worn accelerometry is also low, with many studies averaging less than 12 h of wear time per day [15,16], while relying on arbitrary thresholds for determining both non-wear time and the number of monitoring hours required for a valid day during the data reduction process [17].

Other accelerometer attachment methods have been developed and tested with the aim of generating higher participant compliance than belt-mounted systems. The activPAL accelerometer/inclinometer, which is typically fastened to the thigh using medical dressing, has been utilized in a number of studies to differentiate between sitting and standing/walking, although it is rarely used to estimate the various physical activity intensities quantified by belt-mounted accelerometers. It is possible that the pairing of thigh- and trunk-mounted accelerometers may offer the ‘best of both worlds’, allowing postural transitions to be measured along with traditional activity intensity estimation. Bassett et al. [18] demonstrated the potential of utilizing paired activPAL accelerometers, one taped to the thigh and the other taped to the rib cage, for accurately classifying lying down, sitting, standing/light activity in the upright position, and stepping in a laboratory setting. While dual-accelerometer systems provide more data with which to estimate body posture, physical activities, and even sleep patterns than individual accelerometers, it is possible that the increased participant burden may lead to a reduction in compliance. Wrist-worn accelerometers, which are becoming increasingly popular in the field of physical activity assessment, appear to increase participant compliance but are limited in their ability to differentiate between postural states.

The Axivity AX3 is a 3-axis logging accelerometer that can measure continuous time-series movement data at a wide range of sample rates and durations. When pairs of these waterproof devices are attached directly to the thigh and lower back, they are capable of both physical activity estimation (intensity, duration, and frequency) and posture detection (sitting, standing, and lying). An inbuilt skin temperature sensor removes the ambiguity surrounding sensor use, facilitating estimates of compliance. Unlike most other accelerometer technology, the AX3 is open source, which enables the researcher to have substantial control over the configuration and processing of data, and is not restricted by the manufacturer’s proprietary software and algorithms. This also means, however, that a significant level of technological expertise and computer programming knowledge is required. Nonetheless, these units represent a potential improvement over some more commonly used measurement technologies, particularly for the development of 24 h behavioural profiles. A Danish research group have demonstrated the promise of a dual-AX3 system for assessing physical activity and sedentary behaviour in children, reporting five full days of data (67.9–74.8%, depending on attachment site) and have been instrumental in developing AX3 data collection and processing protocols which can be adopted elsewhere [19,20]. This system has since been used to evaluate the effects of school-based interventions [21,22], while a single-unit, wrist-worn AX3 system has been developed for the UK Biobank study [23], the world’s largest dataset that includes device-based physical activity estimates. However, 24 h compliance with a dual-AX3 system able to differentiate between activity types in adults has yet to be examined or compared with compliance in children.

It is clear that our ability to objectively measure and classify behavioural compositions is presently limited. This forms a significant barrier to the uptake and utilisation of time-use data in behavioural research and inhibits the progression of this emerging field. We believe the next step is to develop cost-effective, multi-accelerometer measurement systems designed to maximise compliance and identify postural transitions over long periods of time. This is in line with the recently developed theoretical framework for Viable Integrative Research in Time-Use Epidemiology (VIRTUE) [6]. Therefore, the primary aim of this research was to test the wear-time compliance with a dual-sensor system, based on the AX3 accelerometer, for capturing 24 h movement patterns in children and adults. A secondary aim was to provide physical activity researchers with a detailed example of the capabilities of the dual-accelerometer system and how it might add value to future studies.

## 2. Materials and Methods

### 2.1. Study Design

We sourced data from two independent cross-sectional pilot studies, the Growing Up in New Zealand Leading Light *‘Te Roopu Piata’* cohort (November–December 2016) and the Workplace Job Demands pilot study (August–November 2017), henceforth referred to as the child and adult samples, respectively. Funding for Growing Up in New Zealand data collection and coordination was provided by the Social Policy Evaluation and Research Unit (NZ). Funding for Workplace Job Demands data collection and coordination was provided by a Strategic Research Innovation Fund grant by the Auckland University of Technology.

### 2.2. Participants

#### 2.2.1. Child Sample

The Growing Up in New Zealand longitudinal study comprised a cohort of 6853 children born during 2009 and 2010 [24]. Participants were recruited before their birth, via enrolment of their pregnant mothers. To be eligible, the mothers had to be residing in a region defined by the Auckland, Counties-Manukau, or Waikato District Health Boards. The 103 children (55.8% male) aged 8 years in the present study were members of the Leading Light ‘*Te Roopu Piata*’ subgroup, recruited approximately 6 months before the main cohort of participants, from the same regions. The Leading Light *‘Te Roopu Piata’* participants played an essential role in the assessment of the measurement tools used in the main cohort. The data presented in this paper were collected from the most recent assessment wave; the first wave that implemented accelerometer-based measurement to assess movement profiles. Informed written consent was obtained from the parent or guardian of each child. The study was conducted in accordance with the Declaration of Helsinki, and the protocol was approved by the NZ Health and Disability Ethics Committee (NTY/08/06/055AM03).

#### 2.2.2. Adult Sample

The *Workplace Job Demands* pilot study recruited 83 Air New Zealand employees (65.9% male) aged 20 to 60 years (median age category: 40–44 years). Employees from three work units (corporate, maintenance, and engineering) were eligible for participation if they worked for Air New Zealand in Auckland. Written informed consent was received from each participant. The study was conducted in accordance with the Declaration of Helsinki and the protocol was approved by the Auckland University of Technology Ethics Committee (17/109).

### 2.3. Instruments

The instrument of interest in this study was the Axivity AX3 accelerometer. The AX3 is a small (23 × 32.5 × 7.6 mm; 11 g) waterproof (IPX8), 3-axis accelerometer capable of logging acceleration data at 100 Hz for 14 days (512 MB flash memory). It incorporates a real time quartz clock for precise time keeping and skin temperature sensor (1 Hz) to facilitate monitoring of wear time. Detailed specifications can be found on the Axivity website (http://axivity.com/product/ax3).

**Figure 1:**
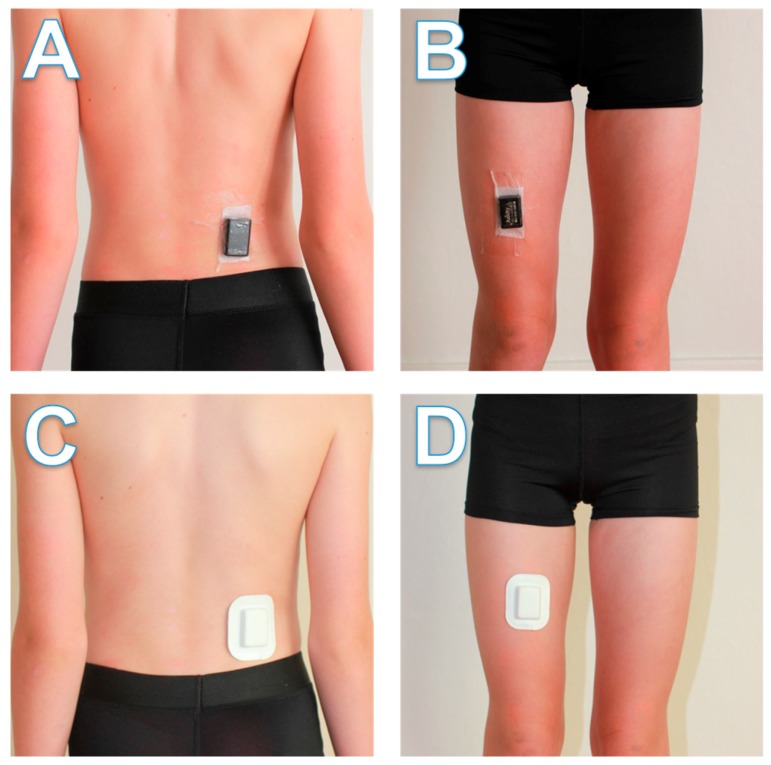
Attachment locations for the paired Axivity AX3 accelerometers. (**A**) Back sensor fastened with medical dressing; (**B**) Thigh sensor fastened with medical dressing; (**C**) Back sensor fastened with foam pouch; (**D**) Thigh sensor fastened with foam pouch.

For all child participants and 19.3% of adult participants (due to a delay in the development and delivery of the pouches used in the second method), one AX3 unit was fastened to the right side of the lower back, above the upper point of the posterior iliac crest, with the other fastened to the front of the right thigh between the hip and knee joints (Figure 1). Attachment sites were first prepped with an alcohol wipe and a 30 × 50 mm section of Fixomull dressing (BSN Medical, Hamburg, Germany) with a strip of double-sided tape. The units were then oriented identically (positive *x*-axis pointing downward, negative *z*-axis pointing forward) and placed on top of the dressing, before being secured to the site with Tegaderm transparent film (1626 W; 102 mm × 121 mm; 3 M, St. Paul, MN, USA). The thigh unit was attached while the participant was standing and the back unit while the participant was seated and slightly leaning forward. On the remaining adult participants (80.7%), we tested new custom-designed foam pouches intended to minimise skin irritation, consumable costs, and researcher burden (Figure 1). The 51 × 65 × 9 mm pouches have an adhesive surface that could be fastened directly to participants’ skin without any additional medical film. Attachment sites were prepared with an alcohol wipe and then units were attached at the same sites and orientation as the previous method. Child and adult participants were instructed to wear the units at all times (including sleeping, showering, and swimming) for a period of seven consecutive days. Spare dressing and instructions for reattachment were provided in the event a unit became detached during the assessment period. Participants were also instructed to remove both units immediately should any skin irritation or discomfort occur.

### 2.4. Procedures

#### 2.4.1. Child Sample

A face-to-face interview with each child (and their parents) was conducted by trained interviewers, where a series of questionnaires, anthropometric variables, and behavioural tasks were assessed. Accelerometer attachment was performed as part of this interview session. The interviewer first explained the accelerometer monitoring protocol to the child and their parent. Participants could opt out of the accelerometer component if they wished, particularly if the child had sensitive skin or a history of skin irritation. The accelerometers were placed on the child’s dominant leg and corresponding side of their back (i.e., both on same side of the body) using the procedure described above. Participants were instructed to wear the units at all times for 7 days. After removal of the units, participants returned the accelerometers to the research team via a pre-paid courier bag that was left with them during the interview.

#### 2.4.2. Adult Sample

All adult participants attended an appointment with a researcher at their worksite during work hours. As per the attachment procedures described above, the researcher attached the thigh unit while the participant was standing and the back unit while the participant was seated and slightly leaning forward. The participants were instructed to wear the accelerometers at all times (including water activities and sleep) for seven consecutive days. Each participant completed a short pre-study survey and was asked to keep a log during the monitoring period of their waking and sleeping times, commuting time, and working times. In addition, participants were asked to report on the log sheet any discomfort or removal of units. On the eighth day, participants were instructed to remove both units and return them to a collection box at the worksite, with the log sheet.

### 2.5. Data Treatment

Data treatment occurred across four sequential phases: downloading, conversion, synchronisation and wear time detection, and activity/posture classification. The AX3 accelerometer stores data internally in a binary packed format known as Continuous Wave Accelerometer (CWA) format. These raw data were downloaded through the Open Movement software (OMGUI) and stored as CWA files. Next, a MATLAB script (MathWorks, Natick, MA, USA) provided by Open Movement was used to convert these raw data into .mat files, which are compatible with MATLAB. During this conversion process the acceleration data were resampled to 100 Hz using a polyphase anti-aliasing filter, while the temperature data were resampled at 1 Hz. This was performed because the actual sensor sample rates fluctuated between approximately 94–104 Hz (acceleration) and 1.5–2.0 Hz (temperature).

**Figure 2:**
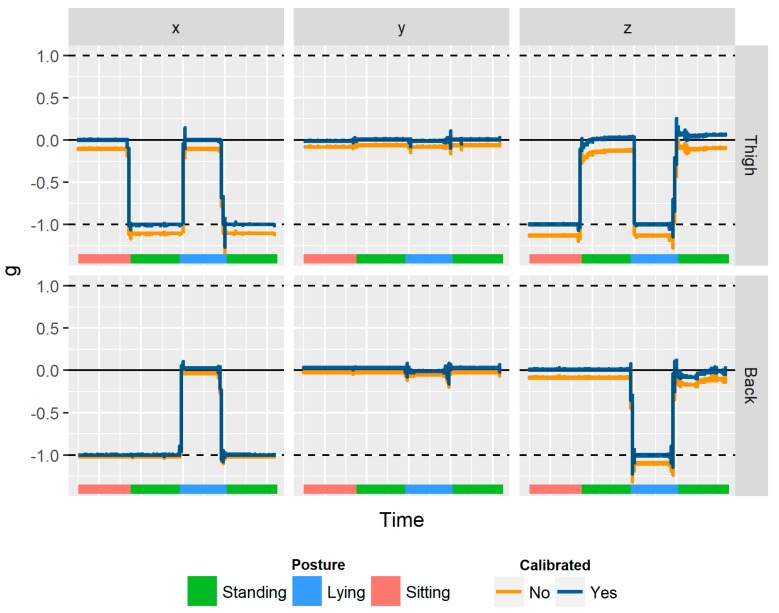
Example of pre- and post-calibration motion sensor outputs for paired Axivity AX3 accelerometers at three different postural states (standing, lying, sitting).

We noticed the recorded acceleration values can differ across sensors when placed under the same conditions, thus calibration of device data is critical. During the conversion process the individual *x*, *y*, and *z* axis values were adjusted using an offset and scale calculated from a simple calibration trial (Figure 2). Each sensor was left completely static for 30 s in six different orientations. These six positions placed each of the three axes in positive and negative gravity, where the measured acceleration would ideally equal ±1 g. The centre point of each positive and negative axis pair was adjusted to reside at 0 g (i.e., the offset) before being multiplied by a scale, so the minima and maxima resided at ±1 g.

**Figure 3:**
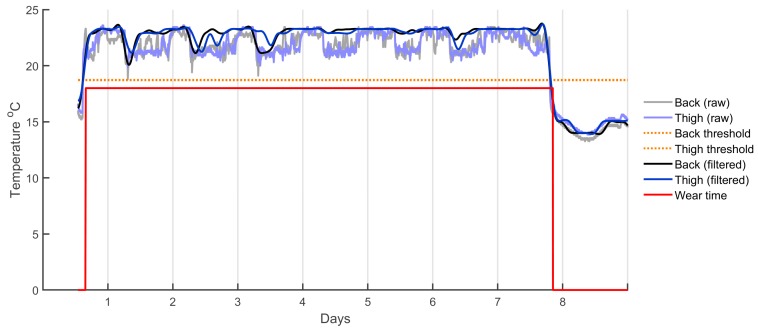
Example of temperature sensor outputs and wear time thresholds for paired Axivity AX3 accelerometers.

Once the data were downloaded, resampled, and calibrated, wear time detection was performed. In this study, wear time was classified as both sensors being worn concurrently. As such, the back and thigh data streams were synchronised and trimmed by finding the earliest and last common timestamps of valid wear time across both sensors. Wear time was detected based on temperature readings across time—as shown in Figure 3—with each sensor processed individually as temperature could vary across sensors. First, a dynamic temperature threshold was set by finding the mean temperature above and below 18 °C (selected as a suitable starting point after visual inspection of the data) and the midpoint between these two values was used as the threshold. Temperature readings above this threshold were considered wear time, while temperatures below were considered non-wear. However, a temperature band hysteresis (an on-off temperature deferential that favours the previous state) of ±2 °C was implemented to prevent the wear state from rapidly toggling on and off due to small fluctuations in temperature. The wear state was only changed when a significant change in temperature was observed, a process implemented to deal with the normal temperature fluctuations across a day. Although these temperatures work well in New Zealand, they may not perform as well in environments where ambient temperature is close to or higher than surface skin temperature.

A simplistic approach to posture detection was taken for demonstration purposes but these detection parameters still require validation. The orientation of both sensors was considered to classify each one-second epoch as standing, sitting, or lying down. Acceleration in the vertical axis (in this case the *x*-axis) was used to differentiate between each postural position. Periods of upright activities (including walking) were classified when both the back and the thigh sensors were vertical (i.e., *x*-axis is close to 1 g). Sitting-based activities were classified when the thigh sensor was horizontal (i.e., *x*-axis close to 0 g) and the back sensor was vertical. Lying was considered when both sensors were horizontal. Due to the variability in postural positions observed during testing, a range was used (i.e., 0.7–1 g for vertical, −0.5–0.5 for horizontal). However, it should be noted that these thresholds were used as a ‘proof of concept’ and require further validation.

### 2.6. Data Analysis

Due to the skewed distribution, valid hours of wear time were presented as mean, 5% trimmed mean, median, and interquartile range (IQR). Comparisons of wear time between samples and attachment methods (medical tape vs. pouch) were made using Mann-Whitney *U* tests. Comparisons of wear time between attachment sites were made using Wilcoxon signed-rank tests. The percentage of time spent standing/walking, sitting, and lying were presented as mean and standard deviation, and between-group comparisons were made using independent-samples *t*-tests. All statistical analysis was completed using R 3.4.3 (R Foundation for Statistical Computing, Vienna, Austria).

## 3. Results

**Figure 4:**
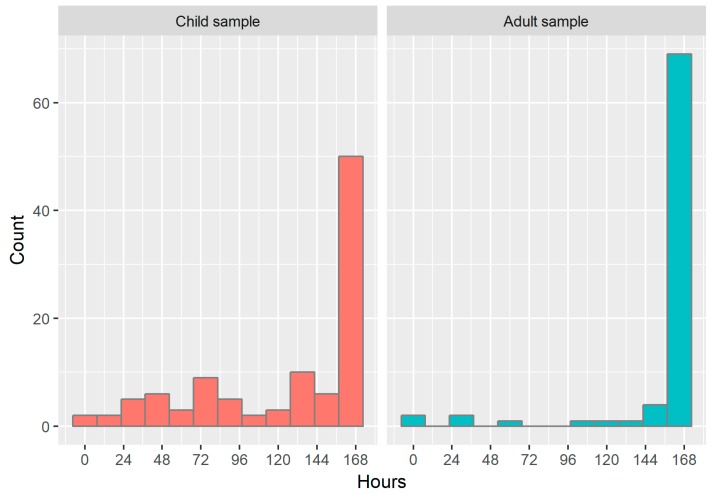
Histogram of valid hours of wear time for paired Axivity AX3 accelerometers in children and adults.

Figure 4 shows a histogram of the hours of valid wear time observed for both samples out of a total possible wear time of 168 h (24 h × 7 d). The median (IQR) wear time in children was 160 (67.0) h and 165 (79.3) h for back and thigh placement, respectively. Wear time was significantly higher (*p* < 0.001 for both sites) and less variable in adults, with a median (IQR) for back and thigh placement of 168 (1.00) and 168 (0) h, respectively. There were no significant differences in wear time between attachment sites for children (*p* = 0.527); however, in adults, wear time for thigh sensors was significantly higher than for back sensors (mean difference = 2.30 h; *p* = 0.034). Significant differences were also observed between attachment methods in the adult sample: the 25th and 75th percentiles for participants with the foam pouches were 167 h and 168 h (respectively), compared with 146 h and 168 h for participants with medical dressing (*p* = 0.045; median = 168 h for both groups).

**Figure 5:**
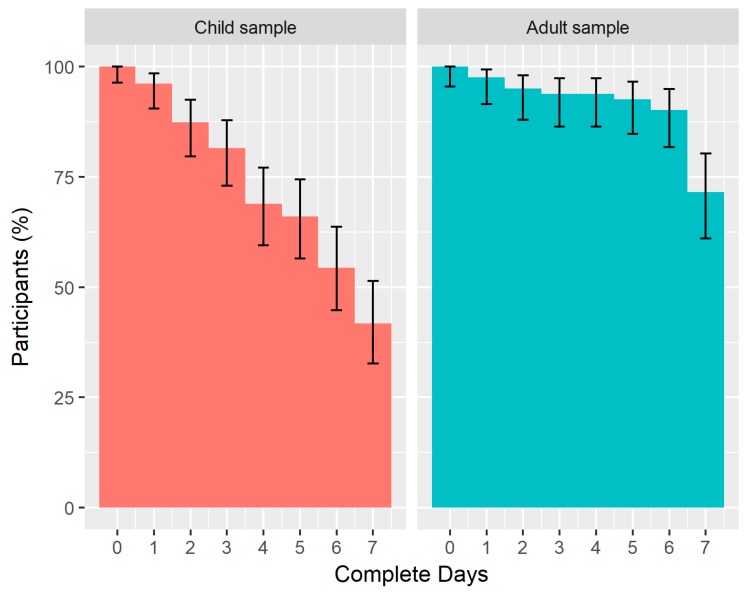
Number of complete 24 h days of data obtained from paired Axivity AX3 accelerometers in children and adults. Error bars represent 95% binomial proportion confidence intervals, calculated using the *binconf* function in the *Hmisc* R package.

Figure 5 shows a reverse cumulative distribution plot of the number of complete 24 h days (out of a total of seven possible complete days) obtained from the child and adult samples. The median number of complete days (IQR) was 6.53 (3.63) days for children and 7.00 (0.05) for adults, with a higher proportion of adults achieving the maximum number of complete days (71.6% compared with 41.7% in children). The biggest percentage decrease occurred between 3 and 4 days in children (12.6% decrease) and between 6 and 7 days in adults (18.5% decrease).

Table 1 contains the number of problems reported by the adult participants in relation to skin irritation and the adhesion of the sensors and the corresponding associations with wear time (comparable data were not collected for the child sample). Compared to participants with medical dressing, participants wearing foam pouches reported a greater number of issues relating to irritation and adhesion. Almost one in three participants wearing foam pouches reported some form of skin irritation, despite exhibiting higher levels of wear time than participants with medical dressing. Reporting skin irritation was associated with a 10 h decrease in mean wear time across the 168 h assessment period (5.95%); in contrast, reporting adhesion issues was associated with a 15 h increase in mean wear time (8.93%).

Table 2 shows comparisons between the basic postural metrics obtained from the paired AX3 accelerometers in both samples. In a typical 24 h day, children and adults in this study spent approximately one quarter of their time standing or walking. The greatest proportion of time was spent lying, although children averaged 8.04% more lying time than adults (*p* < 0.001). Conversely, adults spent 9.01% more time sitting than children (*p* < 0.001), with no significant differences in the proportion of time spent standing/walking.

## 4. Discussion

The aim of this study was to examine compliance with a dual-accelerometer protocol for monitoring 24 h movement patterns in children and adults. It built on previous work by Schneller and colleagues that implemented an equivalent dual-accelerometer system to measure physical activity in Danish youth [19,21,22]. The findings from the present study indicated that this system provided continuous activity data in free-living children and adults, with good participant compliance in the both age groups. We were also able to generate time spent in various states of activity and to demonstrate the utilisation of an integrated temperature sensor for determining compliance, which negated the need for arbitrary wear time thresholds that can lead to classification error [15,17]. We therefore believe that this new method represents a step forward in our ability to continuously monitor a range of physical behaviours in free-living populations.

One of the anticipated outcomes was the difference in overall wear time and complete days of data between children and adults. The full complement of seven complete days was obtained for 41.8% of our child sample (71.6% of adults), although 66.0% of children completed five full days (92.6% of adults), often cited as the minimum number of days required for a reliable estimate of physical activity in children [25]. In their sample of 903 Danish children aged 11.0 ± 1.0 years, Schneller et al. [19] observed a slightly higher percentage that achieved five full days of dual-AX3 accelerometer monitoring (67.9–74.8%, depending on attachment site), which may be explained by the higher mean age of the sample. It is difficult to make similar comparisons with studies using traditional belt-mounted accelerometers: the threshold for a complete day in the present study was 24 h, whereas most studies define 8-12 h of wear time as a complete day [15] and belt-mounted accelerometers are usually not intended to be worn while asleep. A notable exception was the International Study of Childhood Obesity, Lifestyle, and the Environment (ISCOLE), which asked child participants to wear a belt-mounted accelerometer for 24 h, even while asleep [26]. The latter study reported significant improvements to compliance characteristics compared to the 2003–2006 National Health and Nutrition Examination Survey (NHANES), including an average of 61.8 more minutes of waking wear time each day and 22.6 out of 24 h possible wear time, suggesting that it may only be waking hours that affect compliance. Of the 648 children who wore accelerometers in the ISCOLE study, 491 (75.8%) provided a sufficient number of valid days to be included in the analysis. Similarly, a recent review of accelerometer non-compliance in children aged 2–18 years reported a mean (baseline) non-compliance rate of 22.7% (range: 1.7–67.8%) in 23 randomised controlled trials that aimed for 2–4 days of 8–12 h wear time [15]. As a comparison, the non-compliance rate in the present study was 12.6%, 18.5%, and 31.1% for 2, 3, and 4 days (24 h), respectively. In other words, it appears that the dual-AX3 system achieves similar or slightly better compliance rates in children than traditional belt-mounted accelerometry methods, with the advantages of obtaining between 12–16 h of additional wear (including water-based activities and sleep time) and enabling complex postural identification.

In the adult sample, the very high compliance rates represent clear improvements over traditional methods. In the 2003–2006 NHANES cycle, only 25% of participants returned seven full days of accelerometer data with at least 10 h of wear time when worn around the waist [16]. Studies using wrist-worn accelerometers tend to report greater compliance due to lower participant burden and reduced potential for irritation (although they detect a different array of activities and postures as paired back and thigh accelerometers). Indeed, compliance improved in the more recent NHANES cycles that used wrist-worn accelerometers: 70–80% of participants, depending on age group, provided at least six days of data with at least 18 h of wear time [16]. The UK Biobank study also demonstrated the utility of wrist-worn AX3 units, with 80.6% of participants providing high-quality acceleration data for at least 150 h out of a scheduled 168 h [23]. Our data show even greater improvements, with 90% of the adult sample providing at least six days of data with at least 24 h of wear time. Further investigation of the 15 participants who wore the accelerometers for six but not seven days revealed that some of these participants removed them only slightly early at the end of seven days (wear time in Day 7: mean = 17.5 ± 6.75 h, min = 2.04 h, max = 23.9 h). In fact, only three participants achieved less than 10 h of wear time on Day 7, which is commonly used as the threshold to register a valid day [27]. It is likely that the higher than usual compliance is due to the adhesive attachment method, which appears more convenient and less burdensome than other attachment systems. Nonetheless, the proliferation of physical activity studies using wrist-worn accelerometers is indicative of a major shift in the field, potentially driven by the desire to obtain reliable 24 h behavioural profiles including accurate sleep data. Indeed, there is a current movement to develop and test algorithms for the detection of sedentary behaviours using wrist-worn accelerometers [28,29]. A three-unit AX3 system, integrating wrist, back, and thigh motion detection, may enable even more complex physical activity recognition and sleep characterisation; however, the ideal combination of sensors for assessing specific behaviours requires further investigation.

A limitation of the present study was that the factors influencing non-compliance were not collected for the child sample. These factors may have explained some of the differences observed in wear time between samples. Almost one in three adult participants wearing foam pouches reported some form of skin irritation; however, as mentioned earlier, wear time was significantly higher in the foam pouch group when compared to participants with medical dressing (*p* = 0.045). An understanding of these issues in children would be valuable for determining the most appropriate method for use in future studies. Another limitation was that the estimates obtained in the sample of Air New Zealand employees are unlikely to be generalisable to the wider population.

## 5. Conclusions

The availability of complex 24 h behavioural data profiles in children and adults is essential given the worldwide movement to embed activity, sitting, and sleep recommendations within a hypothetical 24 h day. The aim of the present study was to determine the utility of a dual-accelerometer system, based on open source AX3 technology, for assessing free-living movement patterns in children and adults. The high levels of compliance we observed, including a high proportion of complete 24 h periods, represent an important progression in our ability to monitor free-living human behaviour. The next step is to develop and test data processing algorithms that enable the prediction of key activities and postures related to physical activity, sedentary behaviour, and sleep.

## Figures and Tables

**Table 1:** Irritation and adhesion problems experienced in the adult sample while wearing dual Axivity AX3 accelerometers.

Problem		Medical Dressing		Foam Pouch		Wear Time (h)				
		*n*	%	*n*	%	*n*	Mean	5% Trimmed Mean	Median	IQR
Irritation	Yes	3	18.8%	21	31.3%	24	147	154	168	0
	No	13	81.3%	46	68.7%	59	157	163	168	1
Adhesion	Yes	0	0%	9	13.4%	9	168	168	168	0
	No	16	100%	58	86.6%	74	153	160	168	3

**Table 2:** Basic physical activity metrics obtained from paired Axivity AX3 accelerometers in children and adults.

Postural State	Child Sample (*n* = 103)		Adult Sample (*n* = 83)	
	Mean ± SD	95% CI	Mean ± SD	95% CI
Standing/walking time (%)	27.0 ± 5.49	25.8, 28.2	26.1 ± 6.77	24.6, 27.6
Sitting time (%)	27.0 ± 6.23	25.7, 28.3	36.0 ± 8.07 *	34.3, 37.7
Lying time (%)	46.0 ± 6.15	44.7, 47.3	38.0 ± 7.23 *	36.4, 69.6

* Significantly different from child sample (*p* < 0.01).
